# Peripheral artery disease and clinical outcomes in patients with atrial fibrillation: A systematic review and meta‐analysis

**DOI:** 10.1002/clc.23678

**Published:** 2021-06-25

**Authors:** Jianfu Zhu, Xiaowei Tan, Jian Zhong Zhou

**Affiliations:** ^1^ Cardiology Department The First Affiliated Hospital of Chongqing Medical University Chongqing China

**Keywords:** atrial fibrillation, cardiovascular events, major bleeding, meta‐analysis, mortality, outcome, peripheral artery disease, stroke

## Abstract

**Background:**

Atrial fibrillation (AF) is the most common cardiac rhythm disturbance and leads to morbidity and mortality. Peripheral artery disease (PAD) is associated with atherosclerotic risk factors and always classified as a vascular disease and deemed to be a bad complication of AF. In patients with AF, the risk and prognostic value of PAD have not been estimated comprehensively.

**Hypothesis:**

PAD is associated with all‐cause mortality, cardiovascular (CV) mortality, and other outcomes in patients with AF.

**Methods:**

We searched PubMed, Embase, and Cochrane Library databases for prospective studies published before April 2021 that provided outcomes data on PAD in confirmed patients with AF. Heterogeneity was estimated using the *I* ^2^ statistic. The fixed‐effects model was used for low to moderate heterogeneity studies, and the random‐effects model was used for high heterogeneity studies.

**Results:**

Eight prospective studies (Newcastle‐Ottawa score range, 7–8) with 39 654 patients were enrolled. We found a significant association between PAD and all‐cause mortality (hazard ratio [HR], 1.42; 95% confidence interval [CI], 1.25–1.62; *p* < .001), CV mortality (HR, 1.64; 95% CI, 1.32–2.05; *p* < .001) and MACE (HR, 1.75; 95% CI, 1.38–2.22; *p* < .001) in patients with AF. No significant relationship was found in major bleeding (HR, 1.22; 95% CI, 0.95–1.57; *p* = 0.118), myocardial infarction (MI) (HR, 2.07; 95% CI, 1.17–3.67; *p* = .038), and stroke (HR, 1.14; 95% CI, 0.87–1.50, *p* = 0.351).

**Conclusions:**

PAD is associated with an increased risk of all‐cause mortality, CV mortality, and MACE in patients with AF. However, no significant association was found with major bleeding, MI, and stroke.

## 1 INTRODUCTION

Atrial fibrillation (AF) is the most common cardiac rhythm disturbance and leads to morbidity and mortality.^1^ The estimated worldwide prevalence of AF in a previous study was 596 per 100 000 men or 373 per 100 000 women, which amounts to approximately 33 million patients.^2^ The incidence of AF increases with age. In Australia, Europe, and the USA, the incidence of AF is >13% in people aged >80 years, which is significantly higher than that in the entire adult cohort (1%–4%).^3^ AF may cause various outcomes. Thromboembolic events, particularly in the brain, are thought to be the most important risk factors for the occurrence of strokes at an advanced age.^4^ In addition, atherosclerotic diseases, such as myocardial infarction (MI) and heart failure, also show increased prevalence in patients with AF.^5^ Several underlying common atherosclerotic diseases and risk factors, including hypertension (HTN), chronic kidney disease, and diabetes mellitus (DM), promote the occurrence of AF.^6^ Therefore, AF not only acts as a risk factor for atherosclerotic events but also as a consequence.

Peripheral artery disease (PAD) is also associated with atherosclerotic risk factors that cause progressive narrowing of the lower extremity arteries.^7^ As an independent item of the CHA2DS2‐VASC score, which is used to guide antithrombotic therapy in patients with AF, it has been closely correlated with the outcomes of AF.^8^ Furthermore, the characteristics of PAD and AF overlap in terms of the pathogenesis, epidemiology, outcomes, and treatment.^9^ Based on previous studies, PAD has always been classified as a vascular disease and deemed to be a bad complication of AF.^10^ In patients with PAD, a meta‐analysis of prospective studies has been performed to prove the prognostic implication of AF.^11^ However, in patients with AF, the risk and prognostic value of PAD have not been assessed comprehensively. Therefore, we performed a systematic review and meta‐analysis of available prospective studies to estimate the risk associated with PAD for primary outcomes, including mortality and stroke, in confirmed patients with AF.

## 2 METHODS

### 2.1 Search strategy

We followed the Meta‐Analysis of Observational Studies in Epidemiology guidelines for this meta‐analysis.^12^ A systematic literature search was performed mainly in PubMed, Embase, and Cochrane Library, and we additionally used Google Scholar to offset any possible omissions. We searched the database using the following medical subject headings: “AF,” “peripheral artery disease,” “peripheral vascular disease,” “ankle brachial index,” “outcome,” and “mortality.” Literature from 1949 to 2021 was searched, and only English articles were included. References of reviews and original studies were manually searched for additional studies. Ethical approval is not applicable for this study.

### 2.2 Study inclusion criteria and primary outcomes

Only prospective cohort studies that included patients diagnosed with AF and assessed the impact of PAD were included. Other inclusion criteria were as follows: (1) mean follow‐up of ≥1 year; (2) absence or presence of PAD as a classification variable in patients diagnosed with AF; and (3) full primary outcome data available including all‐cause mortality, cardiovascular (CV) mortality, major adverse cardiovascular event (MACE) including MI, ischemic attack and CV mortality, major bleeding, MI, and stroke. Clinical outcomes were compared between the patients with and without PAD. The exclusion criteria were: (1) review, systematic review, meta‐analysis, case reports, and conference abstracts; and (2) nonEnglish literature or unpublished literature. For reliability, we included studies in which PAD was defined by the abnormal ABI (ankle brachial index).

### 2.3 Data extraction and quality assessment

Study selection and quality assessment were conducted individually by two investigators, and the differences were resolved by rechecking the source data. The quality of the included studies was assessed using the validated Newcastle‐Ottawa Scale (NOS), which is used to evaluate nonrandomized observational studies. The assessment items include selection, comparability, and outcome, including representativeness of the exposed cohort, selection of the nonexposed cohort, ascertainment of exposure, demonstration that the outcome of interest was not present at the start of the study, comparability of the cohorts on the basis of the design or analysis, assessment of outcome, follow‐up long enough for outcomes to occur, and adequacy of follow‐up of cohorts. Every item represents a score of 1–2, and a higher total score indicates that the quality of the study is better.^13^ Evaluated features of the studies included the author; year of publication; country where the study was performed; number of patients; mean age of the patients; sex; follow‐up duration; ratio of PAD, HTN, coronary artery disease (CAD), DM patients; medical treatment (mainly comprising anticoagulants, antiplatelet agents, statins, and angiotensin‐converting‐enzyme inhibitors/angiotensin‐II receptor blockers [ACEI/ARB]), and the main confounder of each study. The definitions of PAD for each study are shown in a separate table.(Suppl 7)The primary outcomes included all‐cause mortality, CV mortality, MACE, major bleeding, MI, and stroke. All enrolled studies included the adjusted hazard ratio (aHR) of PAD for the primary outcomes.

### 2.4 Statistical analysis

Data analysis was conducted using the statistical software Stata version 16.0 (StataCorp LP, College Station, TX, USA). The aHR and 95% confidence interval (CI) from each study were collected for the analysis. Statistical heterogeneity was estimated using the *I* ^2^ statistic. *I* ^2^ values of 25%, 50%, and 75% indicated low, moderate, and high heterogeneity, respectively.^14^ The fixed‐effects model was used for low to moderate heterogeneity studies, whereas a random‐effects model was used for high heterogeneity studies. Assessment of publication bias was performed using the Begg and Egger test.

## 3 RESULTS

### 3.1 Study selection and baseline characteristics

A total of 2420 studies were identified in an initial tentative search. The first 176 repetitions were excluded. Subsequently, 417 reviews, 519 conference abstracts, and 94 case reports were excluded. The remaining articles were screened by reading the titles and abstracts in detail. After checking for duplicates and evaluating the titles and abstracts, the full text of 109 articles was accessed for a specific evaluation.

**FIGURE 1:**
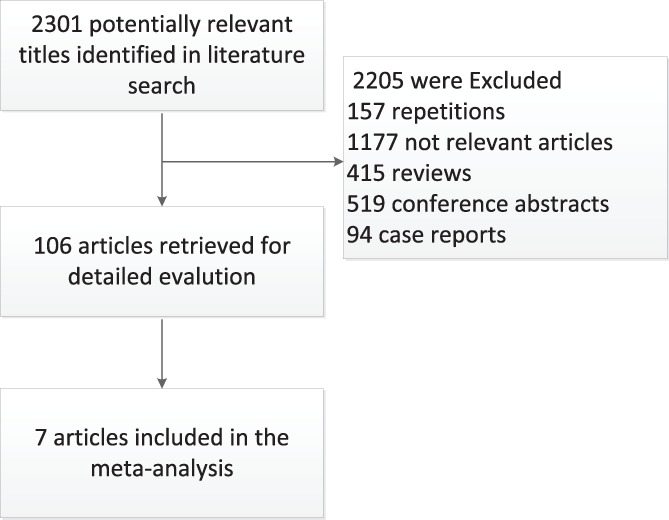
Flow diagram

Finally, only eight eligible prospective studies were included in the analysis^15^, ^16^, ^17^, ^18^, ^19^, ^20^, ^21^, ^22^ (Figure 1), which comprised 39 654 patients with an AF diagnosis. The features of these studies are listed in Table 1. Most studies provided clear percentages or numbers pertaining to the mean age (range 67.1–73.8 years), sex ratio (men 53.7%–64.7%), follow‐up period (range 1–4.8 years), ratio of PAD (range 3.7%–21.3%), ratio of HTN (range 28.8%–87.9%), ratio of CAD (range 18.2%–38.2%), and ratio of DM patients (range 9%–29.4%). Although information regarding the medication use between the PAD and nonPAD groups was collected as far as possible, the treatment variables were not well controlled in some studies. Detailed information on the NOS score is presented in Table 2. The quality of the enrolled articles was medium to high, with a total score of 7–8. The main confounding factors included medications, CAD, MI, and DM, which may have affected the outcomes. All‐cause mortality was provided in five studies, and CV death was assessed in three studies. Furthermore, the hazard ratio of MI was reported in three studies, while stroke was reported in four studies.

**TABLE 1:** Characteristics of studies included in meta‐analysis

Author, Year	Country	No of patients	Mean Age, y	Male, Sex	Follow‐up, y	PAD %	HTN %	CAD %	DM %	Anticoagulants PAD/NonPAD, %	Antiplatelets PAD/NonPAD, %	Statins PAD/NonPAD, %	ACEI/ARB PAD/NonPAD, %	NOS	Confounders
Rasmussen 2011^15^	Denmark	3315	67.1	64.2	4.8	3.7	28.8	NR	9	NR	NR	NR	NR	8	AMI
Hu 2017^17^	International	17980	70	64.7	1.6	4.9	87.9	33.3	25	NR	39/30.6	NR	76.2/71.7	8	Medicine
Proietti 2017^18^	Europe	2975	68.5	59.3	1	11	70.3	32.3	20.8	61.7/59.6	46.3/29.8	49.1/38.0	40.2/37.8	7	CAD, DM, Medicine
Pastori 2018^19^	Italy	1138	72.6	58.7	3	14.8	87.6	22.2	22.4	NR	14.3/19.5	40.5/47.2	NR	7	CAD, Medicine
Inohara 2019^20^	the US	6203	71	58.2	1	8	79.4	25.7	27.2	NR	NR	NR	NR	8	CAD, MI
Vitalis 2020^21^	North America	4060	71	60.7	3.5	6.7	70.8	38.2	20	85.1/84.5	26.3/30.5	NR	38.2/48.6	8	CAD, DM, MI
Vicente 2021	Spain	1956	73.8	56.0	3	6	80.4	18.2	29.4	75.9/73.7	9.9/21.2	54.1/69.5	30.6/34.8	8	CAD, DM, MI, Medicine

Abbreviations: AMI, acute myocardial infarction; ACEI, angiotensin‐converting enzyme inhibitors; ARB, angiotensin receptor blockers; CAD, coronary artery disease; DM diabetes mellitus; HTN, hypertension; MI, myocardial infarction; NR, not reported; PAD, peripheral artery disease.

**TABLE 2:** The quality of the included studies accessed by NOS

	Selection				Comparability	Outcome			
Study	Representativeness of the exposed cohort	Selection of the nonexposed cohort	Ascertainment of exposure	Demonstration that outcome of interest was not present at start of study	Comparability of cohorts on the basis of the design or analysis	Assessment of outcome	Was follow‐up long enough for outcomes to occur	Adequacy of follow up of cohorts	Total score
Rasmussen 2011^15^	*	*	*	*	*	*	*	*	8
Hu 2017^17^	*	*	*	*	*	*	*	*	8
Proietti 2017^18^	*	*	*	*		*	*	*	7
Pastori 2018^19^	*	*	*	*		*	*	*	7
Inohara 2019^20^	*	*	*	*	*	*	*	*	8
Vitalis 2020^21^	*	*	*	*	*	*	*	*	8
Vicente 2021		*	*	*	**	*	*	*	8

* A higher total score indicates that the quality of the study is better.

### 3.2 Association between PAD and all‐cause mortality in patients with AF

**FIGURE 2:**
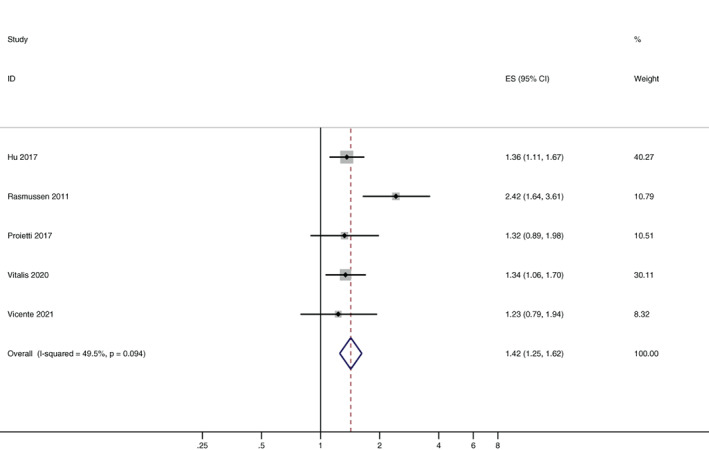
The association between PAD and all‐cause mortality. ES, effect size

There are five articles researched the association between PAD and all‐cause mortality, comprised totally 1735 patients with PAD. There was a significant association between PAD and all‐cause mortality (HR, 1.42; 95% CI, 1.25–1.62, p < .001; Figure 2). Analysis of this outcome showed moderate heterogeneity between the studies (*I* ^2^ = 49.5%).

### 3.3 Subgroup analysis between PAD and all‐cause mortality in patients with AF

We performed a subgroup analysis in age, NOS score, follow‐up duration and sample number (Table 3). Association was significant generally in age ≥ 70 (HR, 1.34; 95% CI, 1.26–2.55; *p* < .001) or < 70 (HR, 1.80; 95% CI, 1.36–2.38; *p* < .001), and in younger subgroup the heterogeneity was much higher (*I* ^2^ = 74.4%). In NOS > 7 subgroup, we also discovered a strong relationship between PAD and all‐cause mortality (HR, 1.44; 95% CI, 1.25–1.65; *p* < .001), with a moderate to high heterogeneity (*I* ^2^ = 61.4%). No relationship was found in NOS ≤7 subgroup (HR, 1.32; 95% CI, 0.89–1.97; *p* = .169) with a low heterogeneity (*I* ^2^ = 0). When it comes to follow‐up duration, a significant association was also revealed whether in ≥2 year (HR, 1.35; 95% CI, 1.13–1.62; *p* < .001) or in <2 year (HR, 1.50; 95% CI, 1.25–1.81; *p* < .001), with low heterogeneity in ≥2 year (*I* ^2^ = 0) and high heterogeneity in <2 year (*I* ^2^ = 72.5%). Finally, a remarkable association was showed in ≥3000 sample number subgroup (HR, 1.46; 95% CI, 1.26–1.69; *p* < .001), with a high heterogeneity (*I* ^2^ = 72.5%). By the way, no significant association was found in <3000 sample number subgroup (HR, 1.28; 95% CI, 0.95–1.73; *p* = 0.103). Heterogeneity (*I* ^2^ = 0) was low between the studies.

**TABLE 3:** Subgroup analysis between PAD and all‐cause mortality in patients with AF

	HR	95% CI	*I*^2^ (%)	*Z*	*p*	Model	Bgger test	Eegger test
All‐cause mortality	1.42	(1.25,1.62)	49.5	5.35	<.001	Fixed model	0.806	0.571
Age								
≥70	1.34	(1.26,2.55)	0	3.91	<.001	Fixed model	0.296	0.018
<70	1.80	(1.36,2.38)	74.4	4.09	<.001	Fixed model	1.000	—
NOS								
>7	1.44	(1.25,1.65)	61.4	5.19	<.001	Fixed model		
≤7	1.32	(0.89,1.97)	0	1.38	0.169	Fixed model		
Follow‐up								
≥2 year	1.35	(1.13,1.62)	0	3.25	.001	Fixed model	1.000	—
>2 year	1.50	(1.25,1.81)	72.5	4.33	<.001	Fixed model	1.000	0.735
Sample number								
≥3000	1.46	(1.26,1.69)	72.5	5.16	<.001	Fixed model	0.296	0.175
<3000	1.28	(0.95,1.73)	0	1.63	0.103	Fixed model	1.000	—

Abbreviations: NOS, Newcastle‐Ottawa Scale; PAD, peripheral artery disease.

### 3.4 Association between PAD and other clinical outcomes in patients with AF

A significant association between PAD and CV death was also revealed for (HR, 1.64; 95% CI, 1.32–2.05; *p* < .001; Suppl 2), with low to moderate heterogeneity (*I* ^2^ = 22.3%). The occurrence of PAD also showed a strong relationship with MACE (HR, 1.75; 95% CI, 1.38–2.22; *p* < .001; Suppl 3), with low heterogeneity (*I* ^2^ = 0). The effect size of each study were shown in Suppl 3 (Pastori 2018: HR, 1.80; 95% CI, 0.95–1.57; Inohara 2019: HR, 1.83; 95% CI, 1.32–2.55; Vicente 2021: HR, 1.55; 95% CI, 0.96–2.52). Only two articles provided the HR for major bleeding, and no statistical difference was found (HR, 1.22; 95% CI, 0.95–1.57; *p* = .118; Suppl 4). The heterogeneity (*I* ^2^ = 0) between studies was low. Since there was a high heterogeneity (*I* ^2^ = 74.6%) among the studies, a random‐effects model was used for data analysis. No significant difference was found between the PAD and nonPAD groups with regard to MI (HR, 2.07; 95% CI, 1.17–3.67; *p* = .038; Suppl 5). Additionally, no significant association was found between PAD and stroke (HR, 1.14; 95% CI, 0.87–1.50, *p* = 0.351; Suppl 6). Heterogeneity (*I* ^2^ = 11.1%) was low between the studies.

### 3.5 Sensitivity analysis and publication bias

We conducted a sensitivity analysis additionally in analysis between PAD and all‐cause mortality. Results of sensitivity analysis revealed that after deletion of any study PAD still related with all‐cause mortality strongly (Suppl 1). Each study was assessed using the Begg and Egger test; no apparent publication bias was found in the range of Begg 0.308–1.000, and range of Egger 0.204–0.967.

## 4 DISCUSSION

A comprehensive evaluation of the prognostic value of PAD in AF was lacking before this meta‐analysis. According to our results, the occurrence of PAD in patients with AF can increase the risk and incidence of several adverse clinical events, including all‐cause mortality, CV death, and MACE, but has no significant impact on the incidence of major bleeding, MI, and stroke. In general, the publication bias in each study using the Begg and Egger test was not significant and no publication bias was found (range of Begg: 0.308–1.000, range of Egger: 0.204–0.967). To ensure the evidence level of the enrolled studies, we rejected reviews, conference abstracts, and case reports, and only prospective cohort studies were selected.

The results of the analysis indicated that PAD promoted poor outcomes in patients with AF, which was in accordance with other unenrolled studies. A cohort analysis derived from the EORP‐AF General Long‐Term Registry, which was excluded from our study due to the absence of respective hazard ratios of vascular diseases, showed a higher incidence of major adverse events and all‐cause death in PAD patients.^23^ A previous nationwide cohort study in Taiwan included 30 203 PAD patients and 552 432 nonPAD patients, and demonstrated a much higher HR of PAD for stroke (PAD, 1.71; nonPAD, 1.29) and CV death (PAD, 3.33; nonPAD, 5.04).^24^

We can assume that a synergistic effect exists between PAD and AF for these clinical outcomes and a previous meta‐analysis also confirmed the adverse impact of AF in patients with PAD.^11^ The mechanisms for the associations between PAD and impacted outcomes are worth investigating. An abnormally higher level of platelet activation has been observed in patients with PAD,^25^ and a significant association between platelet activation and CV risk has been reported by other studies.^26^ Thus, the coexistence of PAD and AF may result in a higher CV risk in all‐cause mortality, CV death, and MACE. Furthermore, peripheral atherosclerosis was reported to increase the inflammatory state,^27^ which can negatively impact the AF. On the other hand, this kind of systematic inflammation relates to the pathophysiology of AF such as fibrosis and thrombogenesis, and may be the cause and consequence of AF concurrently.^28^

On the basis of the outcomes of each study, some conclusions could be made. Stroke is a common AF outcome, and shows a nonsignificant independent association with PAD. This negative result may be based on thrombogenesis as the main pathology of stroke instead of atherosclerosis. Actually, in nonanticoagulated patients, the association increased significantly. Results from the ARISTOTLE trial^17^ and a sub‐group analysis in the AFFIRM study^21^ are also consistent with this phenomenon. Therefore, oral anticoagulation treatment performs remarkably and efficiently in the prevention of stroke as a complication of AF and PAD, and the results of this sub‐group analysis as well as the results of our meta‐analysis indicate that the presence of PAD may be important for guiding anticoagulation treatment.

With regard to major bleeding, the negative result can be explained by the same medical treatment given to the AF and PAD coexistence cohorts. The bleeding risk in patients is always related to medical anticoagulation treatment instead of AF itself or other complications.^29^ Patients who met the treatment criteria were recommended to undergo anticoagulation treatment according to the guidelines,^30^ and demonstrated no association with the occurrence of PAD.

In our study, PAD did not affect the MI incidence in patients with AF, which may have been caused by the separation of the CAD and PAD cohorts among patients, since PAD cannot directly lead to MI. When estimating the risk factors for AF, PAD is always combined with CAD or MI in a group of vascular diseases. The commonly used anticoagulation treatment CHADS2‐VAsc schema also merges the items into one score as an addition to the old CHADS2 schema.^31^ Admittedly, vascular diseases have similar origins and risk factors, and the differences between them deserve further discussion. Recently, a cohort study revealed the distinctive features between PAD and CAD patients, especially between critical limb ischemia and acute coronary syndrome (ACS).^24^ Imparities were also reported in the pathology, epidemiology, and clinical outcomes.^32^, ^33^, ^34^ Even though the atherosclerotic risk and CAD or PAD can sometimes cluster in one patient, individual analysis is necessary for prognostic estimation. Although the results of this study reveal the impact of PAD as an individual vascular factor in patients with AF, the analysis of other factors still remains an issue. A more refined prospective study and relevant analyses are needed.

Due to the negative effects of PAD, appropriate management was necessary in patients with AF. First, diagnosis of PAD depends on the ankle brachial index (ABI), which should be monitored in patients with AF. Moreover, there was limited data on the effects of arrhythmia on the accuracy of the ABI measurement and AF has been confirmed biasing blood pressure measurement,^35^ but a previous study had proved the accuracy of the Doppler measuring method of ABI in patients with AF.^36^ Overall, further related research is needed. In terms of treatment, statins were recommended, and a previous meta‐analysis demonstrated that statins can reduce the incidence of major adverse limb events (MALEs) in patients with PAD.^37^ However, there is insufficient evidence for the use the statins in patients with AF without an ischemic event until a meta‐analysis claimed reduction of all‐cause mortality in patients with AF.^38^ Thus, we advocate a lipid monitor, a more adequate evaluation of dyslipidemia and a radical but appropriate treatment, even in patients with AF who are normolipidemic or without ischemic events. Furthermore, PCSK9 inhibitors (PCSK9i) are more often used in patients with dyslipidemia as an adjuvant therapy of statins or monotherapy, and was proved to be able to significantly reduce the risk of arteriosclerotic CV disease (ASCVD).^39^ On this basis, a previous study claimed that PCSK9i also has a beneficial impact on the incidence of MALE in patients with PAD.^40^ There is no definitive evidence for the use of PCSK9i in patients with AF at present. On the other hand, the cost‐effectiveness of this therapy also limits the clinical application. However, further research on PCSK9i in patients with AF and PAD, even normolipidemic patients with AF, is needed.

Our study has several limitations. First, the sample size of PAD patients was limited in each study, which interfered with the comparison of characteristics between baseline PAD and nonPAD cohorts. In some aspect such as MI, the available data was relatively less, and may lead to a false negative result. Second, we were not able to collect enough data on lipid metabolism, which leads to that subgroup analysis could not be conducted according to different status of lipid metabolism. The lack of data in statins use also caused a considerable restriction in this aspect. Third, the severity of PAD in the included cohorts was unknown. Different types and severities of PAD may influence the outcomes; however, we did not conduct any analysis in this aspect. Further research is needed on the severities of PAD between the clinical outcomes of patients with AF. Moreover, there is an approximately 10‐year time span between the earliest and latest studies. Nonvitamin K antagonist oral anticoagulants are used much more widely and may have caused treatment differences between these studies.

## 5 CONCLUSION

The results of this meta‐analysis showed that PAD is associated with an increased risk of all‐cause mortality, CV mortality, and MACE in patients with AF. However, no significant association was found with major bleeding, MI, and stroke. Although this conclusion was confined to the included studies, it may help in prognostic estimation and establishment of treatment guidelines for AF patients with PAD. Further research is needed to determine the relationship and underlying mechanism between AF and PAD.

## AUTHOR CONTRIBUTIONS

Jianfu Zhu and Jianzhong Zhou contributed to the concept and design of the systematic review. Jianfu Zhu and Xiaowei Tan performed the literature search. Jianfu Zhu performed the statistical analysis, interpretation and writing. All authors contributed to the article and approved the submitted version.

## Supporting information

**Supplementary 1** Sensitivity analysis

Supplementary 2

Supplementary 3

Supplementary 4

Supplementary 5

Supplementary 6

**Supplementary 7** Definition of peripheral artery disease

## Data Availability

The data supporting this meta‐analysis are from previously reported studies and datasets, which have been cited.
