# Supplementary material for: Peripheral artery disease and clinical outcomes in patients with atrial fibrillation: A systematic review and meta‐analysis
Source: Clin Cardiol. 2021 Jun 25;44(8):1050–7. doi: 10.1002/clc.23678 (PMC8364730; doi:10.1002/clc.23678)
Supplement: Supplementary file 1 — Supplementary 1 Sensitivity analysis [file CLC-44-1050-s004.docx]

**Suppl 1 Sensitivity analysis**

| **Study omitted** | **HR** | **95%CI** |
| --- | --- | --- |
| Hu 2017 | 1.47 | 1.24-1.73 |
| Rasmussen 2011 | 1.34 | 1.17-1.53 |
| Proietti 2017 | 1.44 | 1.25-1.65 |
| Vitalis 2020 | 1.46 | 1.25-1.71 |
| Vicente 2021 | 1.44 | 1.26-1.65 |
| Combined | 1.42 | 1.25-1.62 |
