# Supplementary figures and images for: Peripheral artery disease and clinical outcomes in patients with atrial fibrillation: A systematic review and meta‐analysis
Source: Clin Cardiol. 2021 Jun 25;44(8):1050–7. doi: 10.1002/clc.23678 (PMC8364730; doi:10.1002/clc.23678)

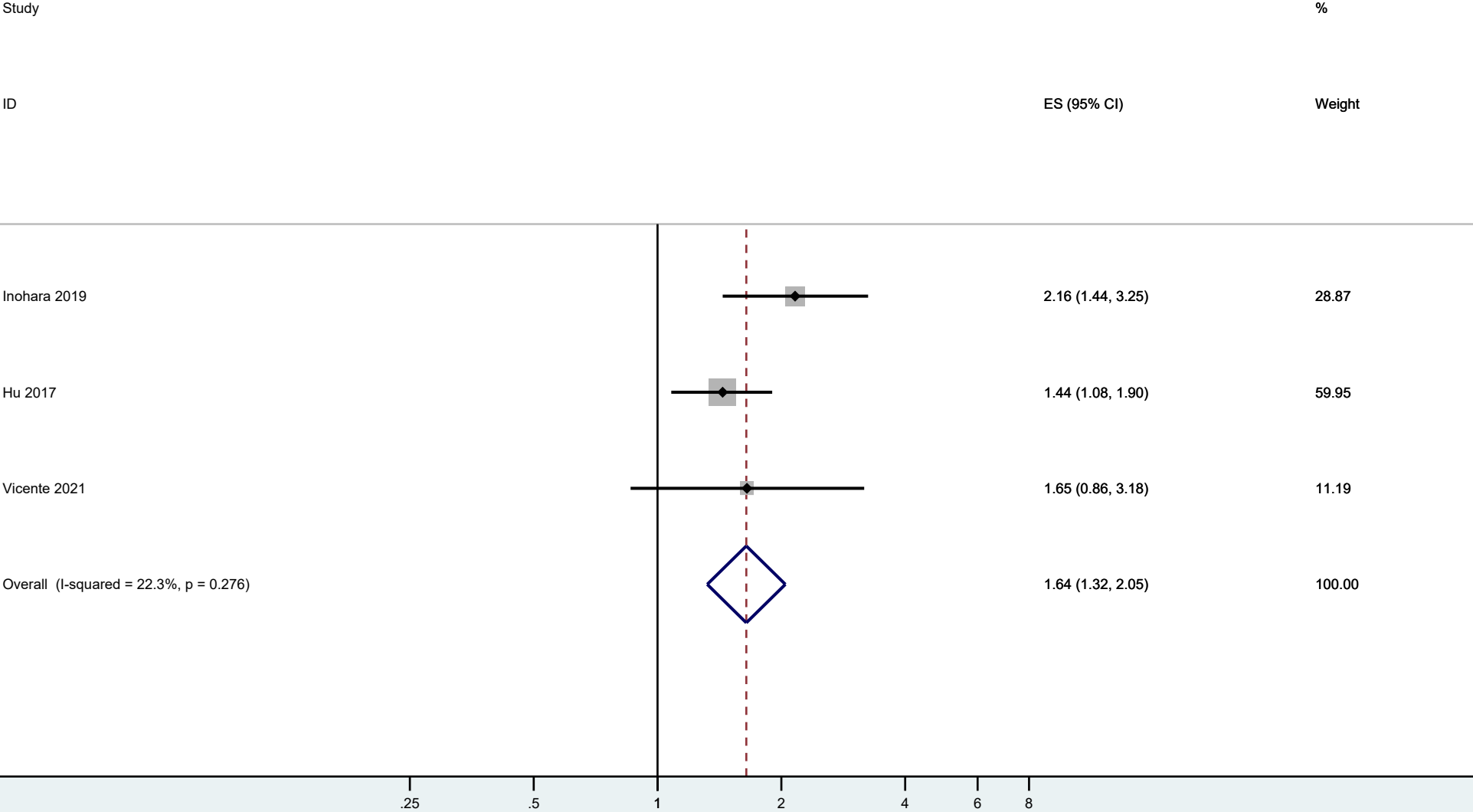

Supplement: Supplementary file 2 — Supplementary 2 [file CLC-44-1050-s003.pdf]

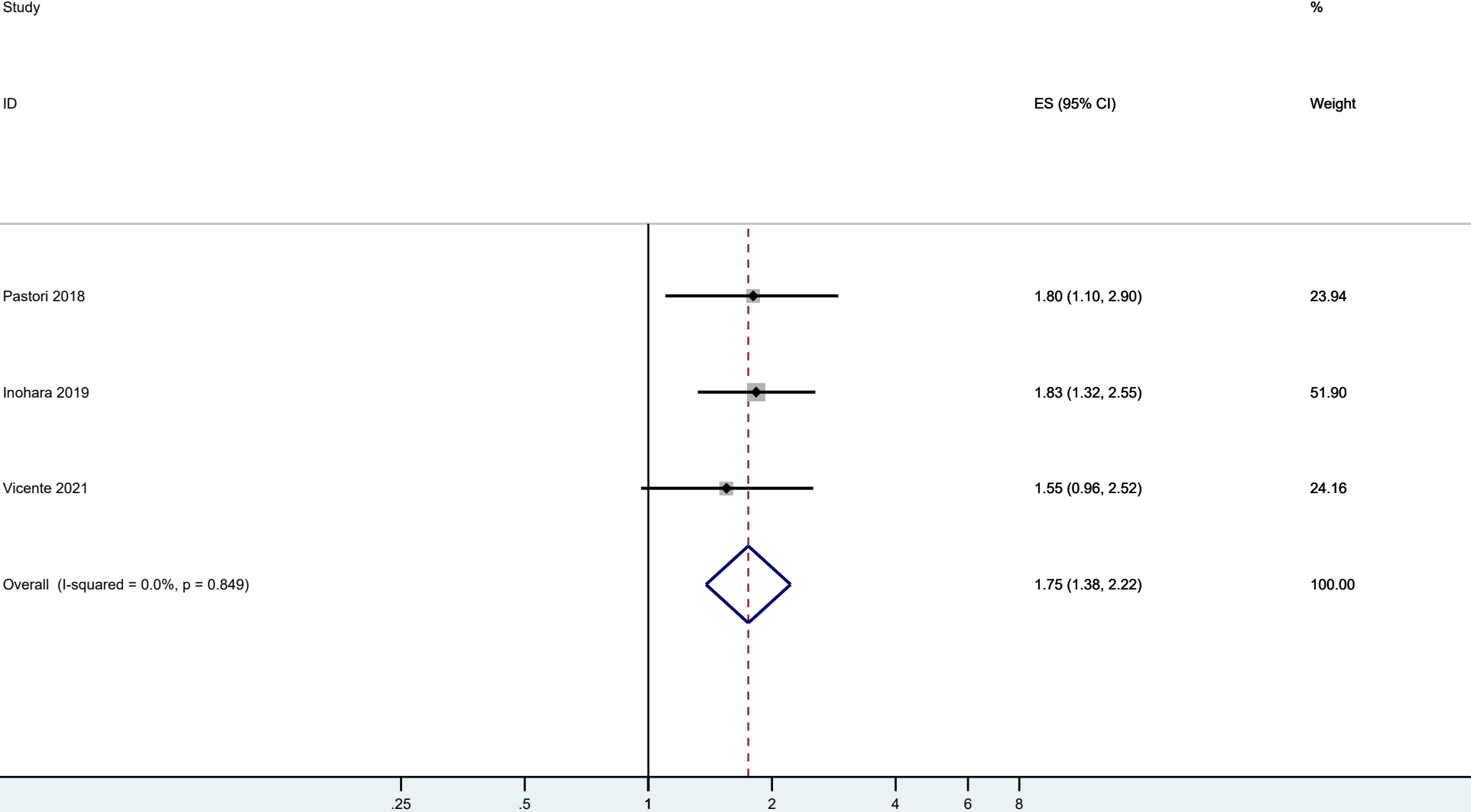

Supplement: Supplementary file 3 — Supplementary 3 [file CLC-44-1050-s001.pdf]

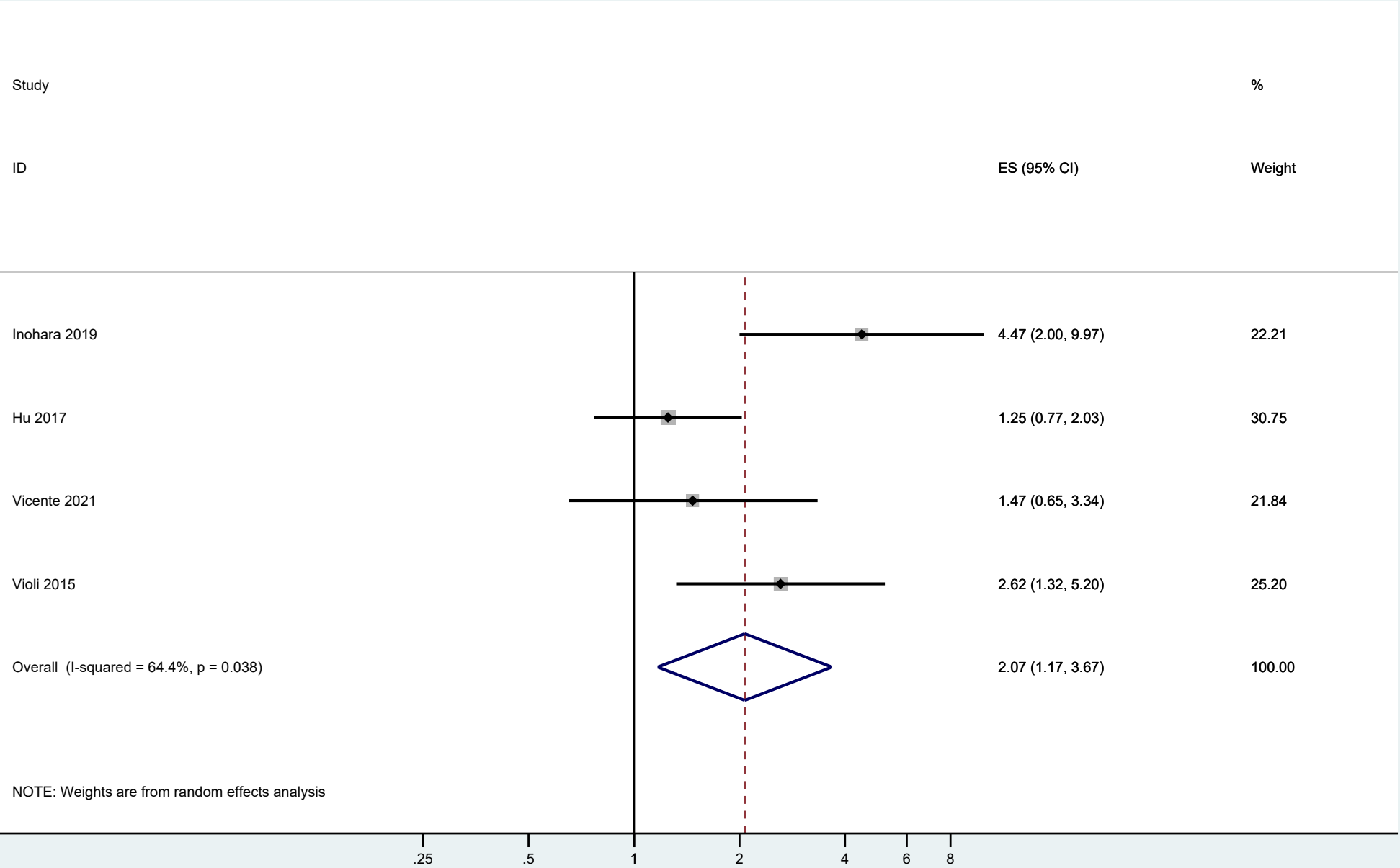

Supplement: Supplementary file 5 — Supplementary 5 [file CLC-44-1050-s002.pdf]

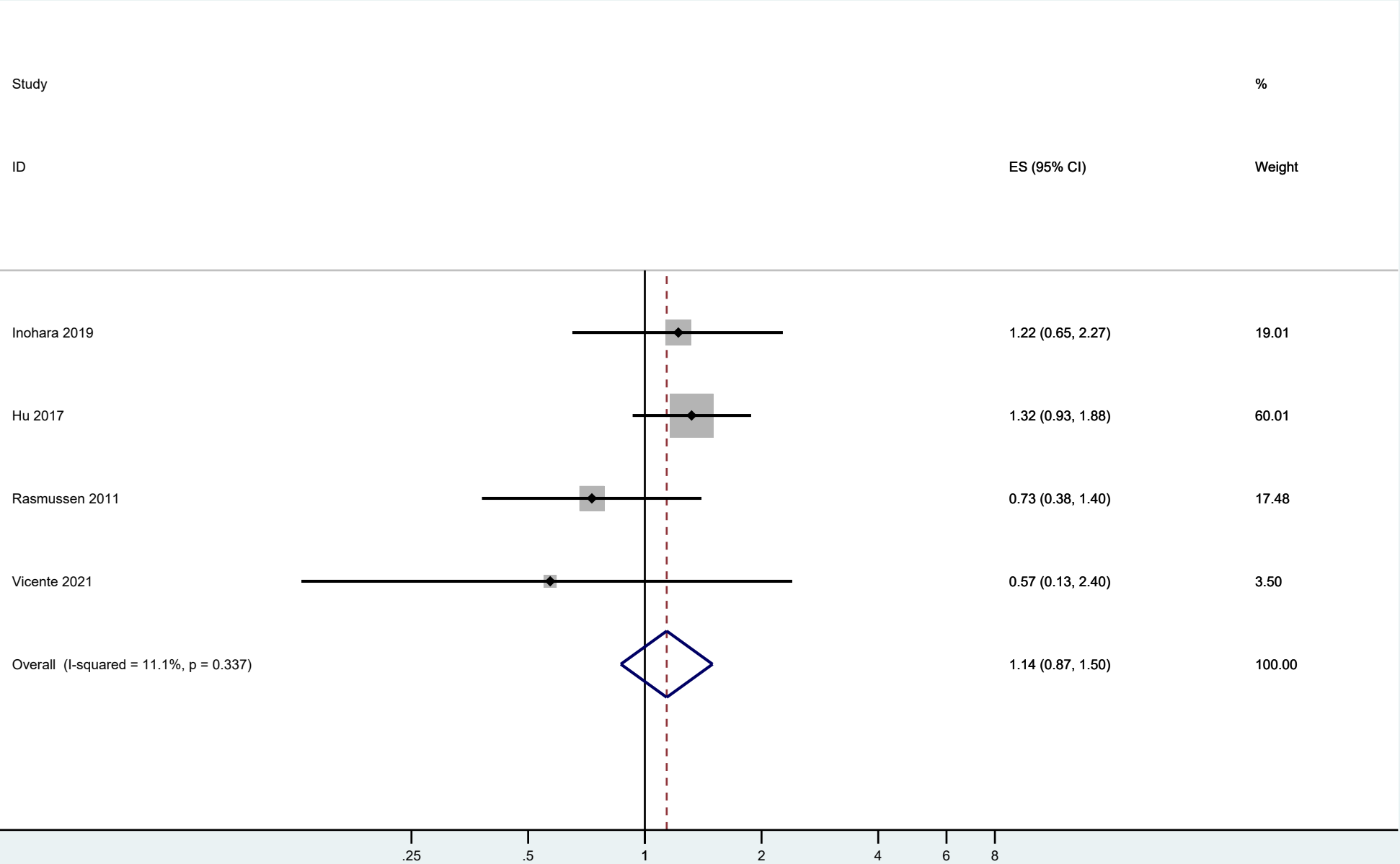

Supplement: Supplementary file 6 — Supplementary 6 [file CLC-44-1050-s007.pdf]
