# Supplementary material for: Peripheral artery disease and clinical outcomes in patients with atrial fibrillation: A systematic review and meta‐analysis
Source: Clin Cardiol. 2021 Jun 25;44(8):1050–7. doi: 10.1002/clc.23678 (PMC8364730; doi:10.1002/clc.23678)
Supplement: Supplementary file 4 — Supplementary 4 [file CLC-44-1050-s005.pdf]

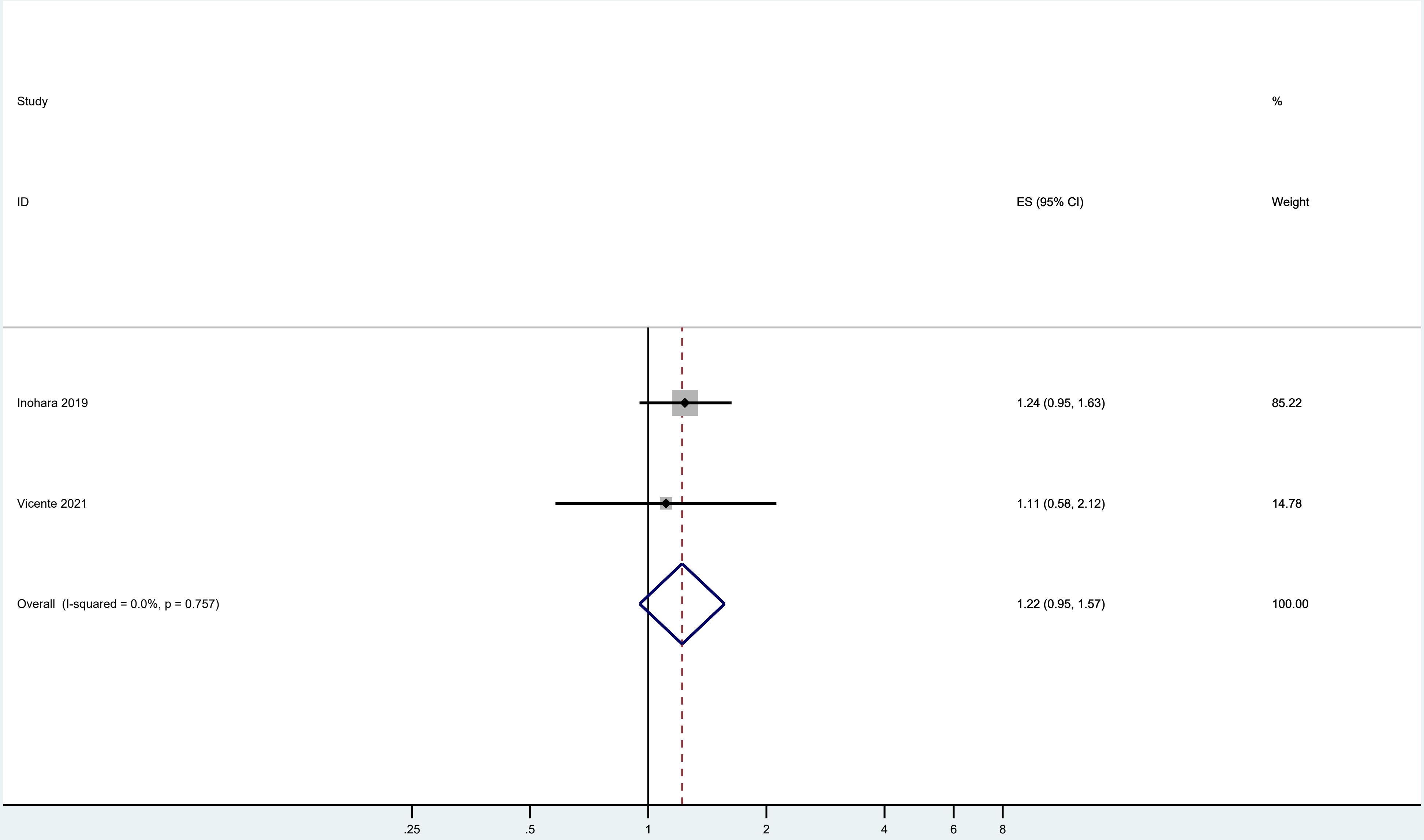

Study
%
ID
ES (95% CI)
Weight
Inohara 2019
1.24 (0.95, 1.63)
85.22
Vicente 2021
1.11 (0.58, 2.12)
14.78
Overall (I-squared = 0.0%, p = 0.757)
1.22 (0.95, 1.57)
100.00
.25
.5
1
2
4
6
8
