# Supplementary material for: Peripheral artery disease and clinical outcomes in patients with atrial fibrillation: A systematic review and meta‐analysis
Source: Clin Cardiol. 2021 Jun 25;44(8):1050–7. doi: 10.1002/clc.23678 (PMC8364730; doi:10.1002/clc.23678)
Supplement: Supplementary file 7 — Supplementary 7 Definition of peripheral artery disease [file CLC-44-1050-s006.pdf]

Suppl 7 Definition of peripheral artery disease

Study	Definition of PAD
Rasmussen 2011	Obstruction of large arteries not within the coronary arteries, aortic arch vasculature or brain
Violi 2015	ABI ≤0.9 measured by using standardized procedures
Hu 2017	The presence of PAD was designated by site investigators and reported on the case report form except carotid artery stenosis and aortic aneurysm
Proietti 2017	A positive history of any of the following: intermittent claudication, previous surgery, percutaneous intervention or thrombosis on abdominal or thoracic aorta, and lower extremity vessels.
Pastori 2018	ABI ≤0.9 measured by using standardized procedures
Inohara 2019	Not mentioned
Vitalis 2020	Site investigators defined, no detail was found
Vicente 2021	Not mentioned

Abbreviation: PAD, peripheral artery disease; ABI, ankle brachial index
